# Experimental and Numerical Investigation into Failure Modes of Tension Angle Members Connected by One Leg

**DOI:** 10.3390/ma14185141

**Published:** 2021-09-07

**Authors:** Edyta Bernatowska, Lucjan Ślęczka

**Affiliations:** Faculty of Civil and Environmental Engineering and Architecture, Rzeszów University of Technology, Poznańska 2, 35-084 Rzeszow, Poland; sleczka@prz.edu.pl

**Keywords:** steel angle members, lap bolted connection, numerical simulations, porous material model, shear lag effect

## Abstract

This paper presents the results of experimental and numerical tests on angle members connected by one leg with a single row of bolts. This study was designed to determine which failure mode governs the resistance of such joints: net section rupture or block tearing rupture. Experimental tests were insufficient to completely identify the failure modes, and it was necessary to conduct numerical simulations. Finite element analysis of steel element resistance based on rupture required advanced material modelling, taking into account ductile initiation and propagation of fractures. This was realised using the Gurson–Tvergaard–Needleman porous material model, which allows for analysis of the joint across the full scope of its behaviour, from unloaded state to failure. Through experimental testing and numerical simulations, both failure mechanisms (net section and block tearing) were examined, and an approach to identify the failure mode was proposed. The obtained results provided experimental and numerical evidence to validate the strength function used in design standards. Finally, the obtained results of the load capacity were compared with the design procedures given in the Eurocode 3′s current and 2021 proposed editions.

## 1. Introduction

Hot-rolled equal and unequal angles are some of the most common structural elements and are usually used as axially loaded tension or compression members. The resistance of these angles when loaded in axial tension strongly depends on the way they are connected. Owing to ease of manufacture and assembly, the most common form of joining is connecting one leg by a single or double row of bolts, while the other, outstanding leg remains unconnected. This results in weakening of the gross cross-section, formation of eccentricities, and the occurrence of a shear lag phenomenon defined as non-uniform tensile stress distribution in the vicinity of a connection, produced by applying local force on the joint.

**Figure 1:**
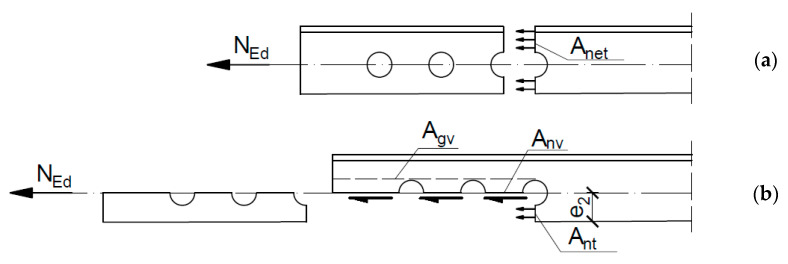
End of the angle weakened by bolt holes; (**a**) the net cross-section area A_net_, (**b**) cross-section areas subjected to tension and shear.

Numerous experimental studies have been conducted to assess the load capacities of such connections. Munse and Chesson [1], Kulak and Wu [2], and Munter and Bouwman [3] conducted tension tests on a wide range of angles. The observed failure modes were described as bearing failure, shear failure of the bolts, and net section failure of the angle. Based on these tests, a few empirical equations were proposed to calculate the net section resistance of tensioned members. Such equations allow the treatment of angles connected by one leg as concentrically loaded without requiring time-consuming determination of bending effects and stress concentration and taking into account the influence of the reduced net cross-sectional area A_net_ on the bending and shear lag effect, as shown in Figure 1a.

Although block shear tearing was first identified in coped beams by Birkemoe and Gilmor [4] in the second half of the 20th century, it quickly became apparent that such failure modes can be decisive in angles connected by one leg. This form of failure occurs due to simultaneous shear failure at the row of bolts along the shear face of the hole group and tensile rupture along the line of bolt holes on the tension face of the bolt group, as shown in Figure 1b. A significant number of investigations have been performed to study the failure mode of angles connected by one leg. Orbison et al. [5] conducted an experimental test of steel angles and tees designed to produce block shear failure and demonstrated the influence of geometrical parameters on resistance. Additional test data were provided by Epstein [6], Ke et al. [7], Jiang et al. [8], and Dhanuskar and Gupta [9]. Some of the studies also covered block shear in angle connections with double-line bolt arrangements [6,9] and the block shear resistance of high-strength steel angle members [7,8]. The block shear resistance of angle connections is generally considered to be a small part of the block shear resistance problem of all bolted connections, and the applied design provisions against block shear failures are mainly calibrated on gusset plates, wide flange sections, tees or coped beam connections.

Today, numerical simulations are an efficient alternative or supplement to experimental tests. Epstein and Chamarajanagar [10] studied the influence of the outstanding leg, shear length, and staggered spacing of fasteners using an elastic–plastic material model. Kulak and Wu [2] made their test more complete by finite element (FE) analysis using nonlinear material and geometry effect behaviour. An extensive FE study on block shear failure of steel tension elements was conducted by Topkaya [11]. Over a thousand nonlinear analyses were performed to identify parameters that influence block shear capacity, but few were dedicated to eccentrically loaded elements, such as angles connected by one leg. Contemporary research work, apart from test results, also includes FE analyses carried out with increasing degrees of complexity [7,8,9]. The modern approach is to use material and geometric nonlinearities and replace simple strain-base criteria to determine the failure load of members using more advanced material damage criteria. In addition, contact conditions that provide proper load transfer to bolts are now usually included. Recently, sophisticated numerical studies of block shear have been carried out that included fracture initiation and propagation, but they were limited to rectangular gusset plate connections or coped beam connections [12,13].

The research on the resistance of angles connected by one leg can be divided into two groups: one deals with the phenomenon of the ultimate resistance of the net cross section, whereas the other deals only with block shear tearing. There is a lack of studies that trace both of these mechanisms in one connection and investigate the boundaries between individual failure modes. Net cross-section failure and block shear tearing in design provisions are described by appropriate individual strength functions. Each function should be statistically evaluated based on test results in which the proper failure mode has occurred. Unfortunately, identification of the failure mode in many experimental tests is difficult or impossible, because both net section tearing and block tearing result in a rupture along the line between the bolt hole edge and angle edge (e_2_ in the direction perpendicular to the acting load: see Figure 1). Numerical analyses may be helpful for solving this problem. Considering the advances in FE modelling, simulations on connections pertaining to the ultimate resistance of the net cross-section or block shear tearing should include the material failure process characterised by ductile fracture. Such sophisticated FE analyses have been performed for block shear fracture of gusset plates and coped beam connections [12,13] but are not yet available for angle connections.

The importance of tension angle members and their joints in different applications is underlined in the construction and maintenance of many metal structures such as masts, transmission line towers, transfer joints in the form of gusset plates [14], and also all-composite modular wall systems [15]. Such wall systems are mainly made of pultruded GFRP profiles, joined by means of metal connectors, often in the form of short angle members. Thus, in many cases metal angle connections significantly influence the behaviour of the whole structure and their sophisticated analysis is crucial for structure integrity.

In this paper, the results of an experimental study on the ultimate resistance of tension angle members connected by one leg using a single row of bolts are presented. They are utilised for validation of numerical modelling, taking into account yielding and ductile crack initiation and predicting fracture. The Gurson–Tvergaard–Needleman (GTN) material model was applied to simulate the material failure process. Comparison of numerical simulations and test results included both global (load–displacement curves, deformations, and longitudinal stresses) and local (sequence of crack initiation and propagation, final fracture profiles) domains.

This study had two objectives considered by authors as novel. The first was to examine the application of a GTN material model to simulate the behaviour of angles connected by one leg whose resistance is based on the ultimate strength of the steel. The use of such an approach provided a better description of phenomena occurring in the failure process. The second objective was to identify the failure modes in such connections and simultaneously identify the relationship between the failure mode and the geometrical parameters describing the connection. Through experimental testing and numerical simulations, both failure mechanisms (net section and block tearing) were examined, and an approach to identify the failure mode was proposed. The obtained results contribute to the experimental and numerical evidence to validate the strength function used in future design provisions [16,17].

## 2. Experimental Program

### 2.1. Test Specimens

**Figure 2:**
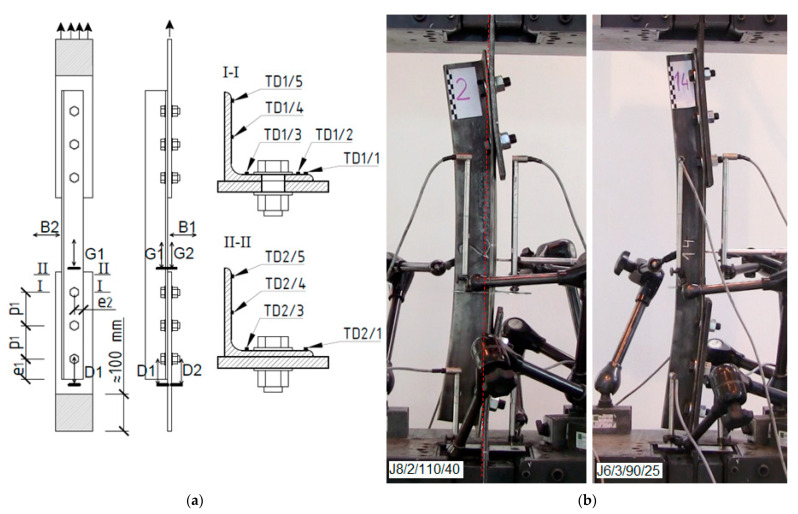
Test specimen: (**a**) placement of measuring devices, (**b**) test set-up.

The experimental testing was performed on 18 equal leg angles connected by various numbers of bolts (*n* = 2–5) to the gusset plates, as shown in Figure 2. The joints were made of two different angles (L80 × 80 × 6 and L60 × 60 × 6) with nominal steel grade S275. The bolts were set in a single-line position. The total length of the angles depended on the number of bolts and the spacing between them, and ranged between 500 and 920 mm.

Fully threaded bolts (M16 and M20 class 8.8 or 10.9) were used. Normal round holes with a nominal clearance of 2.0 mm in accordance with EN 1090-2 [18] were applied to connect the angle and gusset plates at one end of the angle, at which failure was expected. At the other end of the angle, holes with a nominal diameter equal to the shank diameter of the bolt were used. All bolt holes were formed by drilling. Washers were used under both the bolt head and nut in each assembly. Bolted connections were A category according to [19], so only snug-tightening of the bolts was applied.

Gusset plates with cross-section dimensions of 10 mm × 100 mm were made from steel with nominal grade S355. The connections were designed in such a way that failure should occur only in the angle.

The test parameters by which the specimens were differentiated included the dimensions of the hot-rolled angles and the p_1_ (bolt spacing) and e_2_ (edge distance in the direction perpendicular to the acting load) values. The distances p_1_ were taken as 2.5d_0_ or 5d_0_ (these are the limit values for which the β_i_ reduction factor is different when determining the ultimate tensile resistance of an angle in accordance with [19]) or as intermediate values. Two e_2_ values were used for each angle size to determine whether this parameter significantly affected load capacity. A full description of the specimens is provided in Table 1.

### 2.2. Test Set-Up, Instrumentation, and Procedures

All tests were performed using an Instron 1200 kN-J1D testing machine. The investigations consisted of a monotonic tensioning process with displacement control until the destruction of the connection. The applied load and total elongation of the specimens were measured using load cell and displacement transducers built into the testing machine. To measure joint elongation (the local vertical displacements of the angle and gusset plate in one connection), inductive displacement sensors were positioned on both sides of the specimen. Reference points Gi and Di (where i = 1 or 2), used during the measurements, are shown in Figure 2a. In addition, two horizontal sensors, B1 and B2, monitored the displacement of selected points in two directions in the middle of the angle length, as shown in Figure 2. Additionally, for five specimens, strain gauges TDi with working lengths of 10 mm or 5 mm were used in the net cross section of the angle (I-I) and at a short distance from it (II-II) to monitor longitudinal strains. This placed the gauges at 40 mm for connections with M16 bolts or 50 mm for those with M20s.

### 2.3. Test Results

The material properties of the steel angles and plates used in the specimens were determined through tensile coupon tests, according to the process described in [20]. The values obtained are summarised in Table 2.

During the tensioning process, significant bending deformations of the angles and gusset plates were observed. In the final stage of loading, the longitudinal axis of the angles coincided with the direction of the tensile force (Figure 2b).

In 15 of the specimens, rupture of the connected leg was observed in a net cross section at the height of the first internal bolt (cross section I-I in Figure 2a). In three specimens the shear resistance of the bolts was exceeded (marked with letters “BF” in Table 1). However, in one of these (J6/2/90/34), the shear of the bolt was accompanied by simultaneous necking, and fracture initiation was visible near the edge of the bolt hole in net cross section I-I.

**Figure 3:**
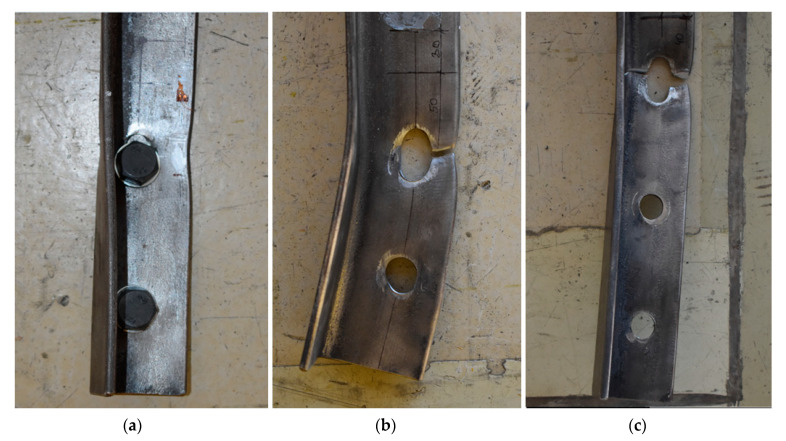
Failure modes of specimens: (**a**) shear of bolts “BF”, specimen J6/2/90/34; (**b**) tearing across the width of connected leg “FI”, specimen J8/2/80/30; (**c**) tearing developed towards outstanding leg “FP”, specimen J6/3/90/25.

Each angle rupture began from necking on the connected leg between the bolt hole and the adjacent edge (on the width described as e_2_). After visible necking occurred, tearing started from the edge of the bolt hole and propagated toward the outer edge of the connected leg. In some elements, fracture was observed only between the bolt hole and the outer edge of the connected leg (marked as “FI” in Table 1). In other elements, after rupture along the e_2_ distance, the fracture propagated towards the outstanding leg (this form of failure was marked as “FP” in Table 1). All failure modes were observed immediately after exceeding the ultimate load F_ult,Ex_ (maximum tensile load registered during a single test) and not after reaching a state of significant elongation associated with a lower load level resulting from the fracture. The observed failure modes are shown in Figure 3.

**Figure 4:**
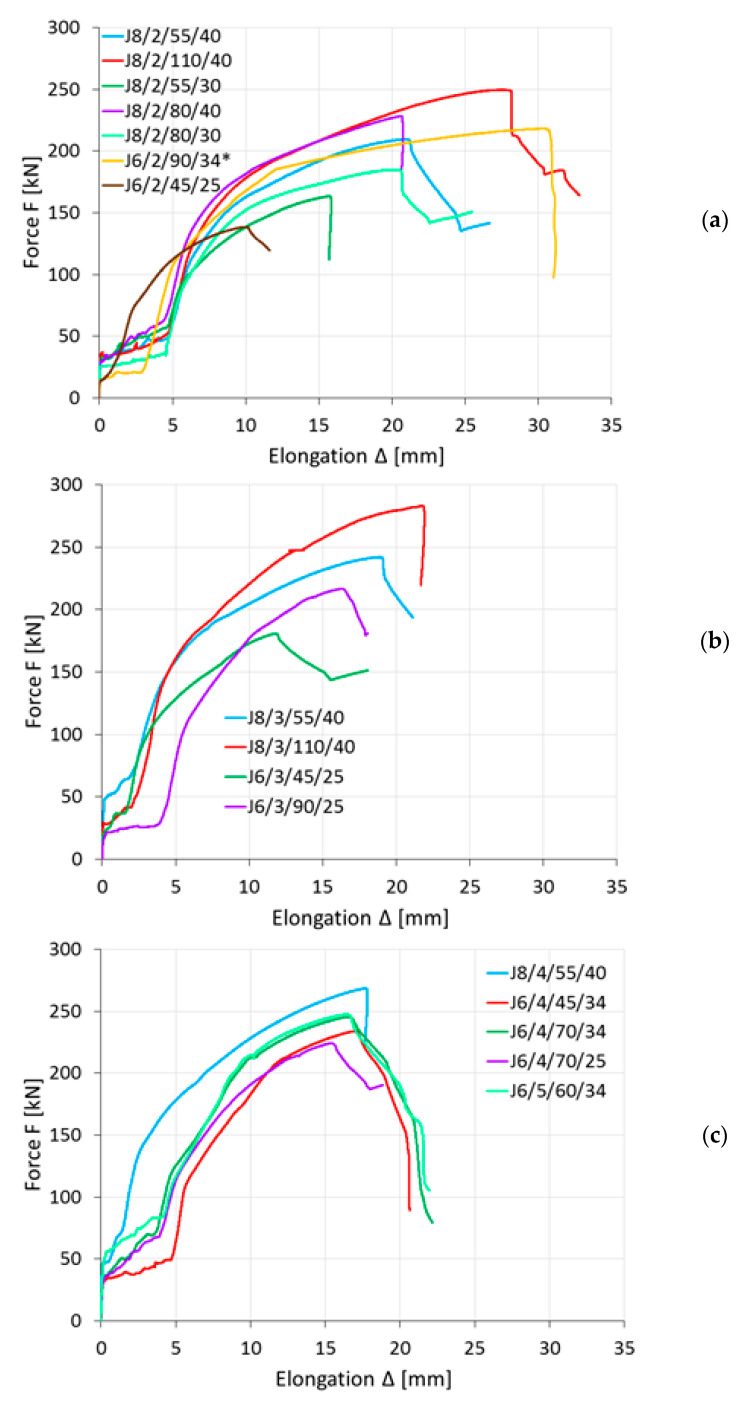
Load–elongation diagrams: (**a**) connections with 2 bolts, (**b**) connections with 3 bolts, (**c**) connections with 4 or more bolts (*: elongation in this case was measured by machine clamps).

The basic data obtained from the experimental tests were the load–elongation (F–Δ) curves, as presented in Figure 4, for elements where angle failure was observed. To define the elongation Δ, the difference in displacement between the reference points Gi and Di was considered. Such measurements were carried out by inductive sensors positioned on both sides of the element (Figure 2) therefore, the Δ value was calculated as the arithmetic mean of the measurements on the left and right sides.

The ultimate tensile resistances F_ult,Ex_ of the specimens (which were the largest load values obtained during the test) are listed in Table 1. For each specimen where angle failure was observed, the efficiency factor of the net cross section U_eff_ was computed as follows:

(1) $$U_{\mathrm{eff}}=\frac{F_{\mathrm{ult},\mathrm{Ex}}}{f_{u}A_{\mathrm{net}}}$$

where f_u_ is the ultimate strength of the angle steel, and A_net_ is the net area of the angle.

This coefficient defines the extent to which the net cross section is utilised in the load transfer. The values of the U_eff_ coefficients are presented in Table 1.

The tests showed the influence of the geometrical parameters on the ultimate resistance of the angles. The obtained results can be effectively used to calibrate the appropriate strength function for block tearing or for net cross-section resistance. However, the experimental tests did not precisely indicate the form of angle failure. In nearly all elements (apart from three specimens where bolt shear was observed) failure appeared through material rupture along the e_2_ distance, which may denote either net cross-section failure (with rupture of the net area of the whole angle A_net_) or block tearing failure (with rupture of net area subjected to tension A_nt_). In a few specimens, the fracture propagated towards the outstanding leg (which can be assumed as the net cross-section failure mode), but this phenomenon can also be explained as the effect of the dynamic process of fracture. To examine the failure process and assign a proper failure mode, it was necessary to conduct numerical simulations.

## 3. Finite Element Analysis

### 3.1. Material Model

Finite element analysis of steel component failure triggered by yielding and ductile crack initiation is a different process compared to the well-established approaches for predicting stability or plastic resistance. Many assessment strategies can be applied, considering the balance between accuracy and cost [21,22]. To model the material failure process, the GTN material model was chosen. This model is intended for use in fracture or damage analysis, and its purpose is to predict ductile crack behaviour through void growth and coalescence [23]. This is a micromechanical, model-based approach that can be effectively employed in structural components without sharp cracks, e.g., in zones of stress concentration around holes. Many studies [24,25,26,27] have shown that the use of the GTN model in the analysis of such structural steel elements yields satisfactory results. However, the application of such an approach to simulate the behaviour of elements in lap bolted joints under tension is limited.

**Figure 5:**
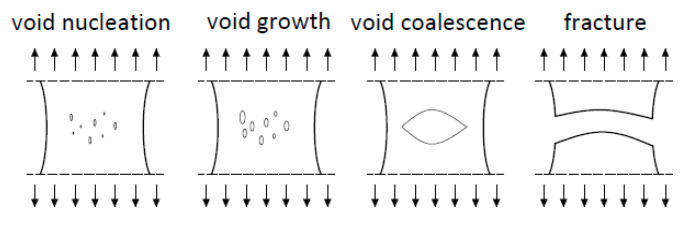
Mechanism of ductile fracture.

For metals, the process of crack initiation and propagation is associated with damage to the material microstructure. These defects, in the form of voids, appear at non-metallic inclusions or precipitates of another phase present in the material. During deformation, the voids grow and connect in a process called coalescence, creating a crack. The ductile fracture mechanism is shown in Figure 5.

Therefore, the material strength, defined by the normal stress σ, is closely related to its damage. The stress value begins to decrease at the moment of void initiation. Void growth causes softening of the material and leads to its destruction when the critical value is reached [26].

According to the GTN material model, the failure criterion is defined as follows:

(2) $$\Phi ={\left(\frac{\sigma_{\mathrm{eff}}}{\sigma_{0}}\right)}^{2}+2q_{1}f^{*}\cos h\left(q_{2}\frac{3\sigma_{m}}{2\sigma_{0}}\right)-\left(1+q_{3}f^{*2}\right)=0$$

where *Φ* is the non-dilatational strain energy; *σ*_eff_ is the effective stress according to the Huber–Mises–Hencky hypothesis; *σ*_0_ is the material yield stress; *σ*_m_ is the hydrostatic pressure (mean stress); *f*^∗^ is the modified void volume fraction, and *q*_i_ is the Tvergaard parameters describing the plastic properties of the material.

The modified void volume fraction *f*^∗^ describes the microstructural properties of the material and is defined as follows:

(3) $$f^{*}=\{\begin{array}{c} f\;\;\;\;\;\mathrm{for}\;\;\;f\leq f_{c},\\ f_{c}+\frac{\bar{f_{F}}-f_{c}}{f_{F}-f_{c}}\left(f-f_{c}\right)\;\;\;\;\mathrm{for}\;f_{c}<f<f_{F}\\ \bar{f_{F}}\;\;\;\mathrm{for}\;\;f\geq f_{F}. \end{array},$$

where *f* is the current void volume fraction; *f*_c_ is the critical void volume fraction at which the void coalescence starts, and *f*_F_ is the void volume fraction corresponding to the complete loss of the material strength at the final separation of the material; $\bar{f_{F}}=\left(q_{1}+\sqrt{q_{1}^{2}-q_{3}}\right)/q_{3}$.

When the material is not subjected to a load, the modified void volume fraction is equal to the initial void volume fraction *f*_0_. This is the basic parameter of the GTN model related to material porosity. The value of *f*_0_ can be determined using Franklin’s formula, as shown in Equation (4), on the basis of the chemical composition [22], where Mn% and S% are the percentages of manganese and sulphur inclusions. These values are determined according to the standard defined in [28] or on the basis of microstructure tests in which the non-metallic inclusions and precipitates of a different phase are counted in relation to the surface of the tested specimen.

(4) $$f_{0}=0.054\left(S\%-\frac{0.001}{\mathrm{Mn}\%}\right)$$

The critical void volume fraction fc is related to the value of *f*_0_ [27], but it can also be determined by fitting the F-Δ curve obtained from numerical simulations to that obtained from experimental tests, or using microscopic photography [29]. The value of *f*_F_ corresponds to the criterion of material failure, and for metals ranges from 0.10–0.20. This can also be experimentally determined [30].

The Tvergaard parameters *q*_i_ influence the strength properties of the material. They are material constants to approximate the real structural behaviour (to better represent the void interaction effect). The optimal proposed values for many metals, including steel, are *q*_1_ = 1.5, *q*_2_ = 1.0 and *q*_3_ = $q_{1}^{2}$ = 2.25.

The damage evolution is described by the following material parameters: *f*_N_, *ε*_N_, and *s*_N_. The first describes the volume fraction of nucleated voids, which for structural steels is assumed to be 0.04. The strain related to formation of new voids is called the mean strain of void nucleation, *ε*_N_. The typical value for structural steel is *ε*_N_ = 0.30. The GTN model assumes a normal distribution of void nucleation strain and a standard deviation *s*_N_ ranging from 0.01–0.10.

Hierarchical validation of the material model used in the analysis was conducted as described in [31]. The final values of the GTN parameters introduced to the Abaqus program, after recognition of the available data and calibration, are presented in Table 3.

### 3.2. Analysis Method

**Figure 6:**
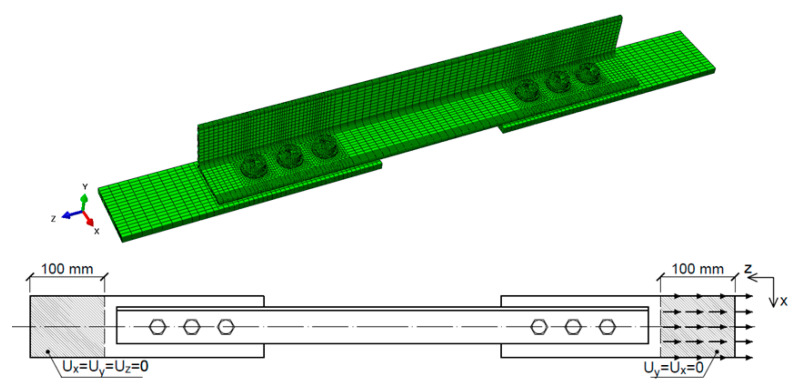
Finite element model and applied boundary conditions.

The commercial software package Abaqus [32] was used to perform the FE analyses. Modelling covered the entire group of tested elements (see Table 1). Only those elements where the failure was visually determined to be caused by bolt shear were omitted. Each FE model consisted of four components: angle, gusset plates, washers, and bolts modelled together with nuts. Part of one gusset plate had blocked displacement in all directions (corresponding to the machine clamps). The load in the z-direction in the form of displacement was applied to the second gusset plate, which had blocked x- and y- displacements on the clenched part. Initially, both washers and bolts were located concentrically with the bolt holes in the angle and gusset plates. Figure 6 presents the view of the complete model with an FE mesh and boundary conditions.

For angles, gusset plates, and washers, a C3D8R type of element was employed, i.e., a three-dimensional hexahedral eight-node linear brick with reduced integration. This type of element has proved to be suitable for simulating lap bolted connections [33,34]. In the vicinity of the bolt holes where stress concentration was expected, the mesh was appropriately dense.

Bolts were built using C3D8T and C3D6T elements, which are eight-node thermally coupled bricks with trilinear displacement and temperature and six-node thermally coupled triangular prisms, respectively. To apply a small clamping force starting from snug-tightened bolts, a vertical thermal deformation method was utilised [35]. The bolt shank was modelled as a smooth cylinder with a diameter equal to the nominal diameter of the bolt for both the M16 and M20 bolts. The bolt thread was not modelled in the shank.

Contact conditions were applied to simulate the interaction between the angles, bolts, washers, and gusset plates. Contact between surfaces was defined using the general contact option [32]. The frictional effects between surfaces were also included by incorporating the classical isotropic Coulomb friction model in the contact definition, with a friction coefficient *μ* equal to 0.1.

For angle elements where fracture was expected, the GTN material model was applied with the parameters shown in Table 3. For the remaining elements (gusset plates, washers, and bolts), an elastic-plastic multilinear material model was implemented. The main material properties, such as yield strength and ultimate strength, were obtained from tests (see Table 2).

Implementation of the GTN porous material model required dynamic explicit analysis with a displacement-based control algorithm.

### 3.3. Comparison of FE Model Results with Tests in Global Terms

**Figure 7:**
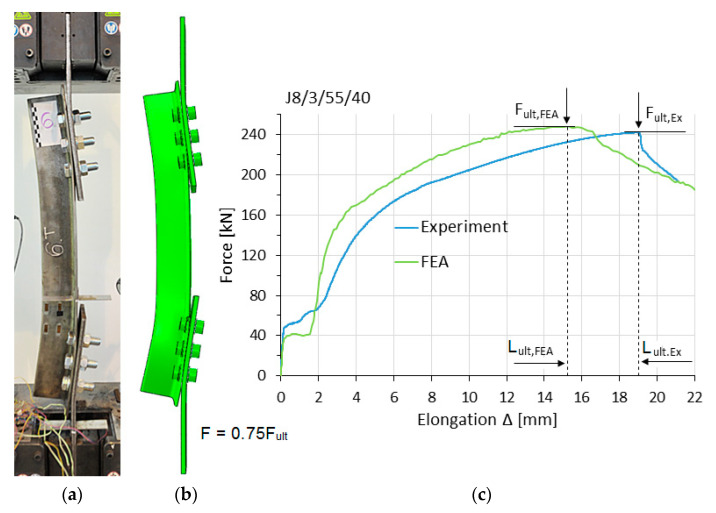
Specimen J8/3/55/40 in the failure phase: (**a**) experiment, (**b**) FE analysis (FEA) model, (**c**) F–Δ curves.

**Figure 8:**
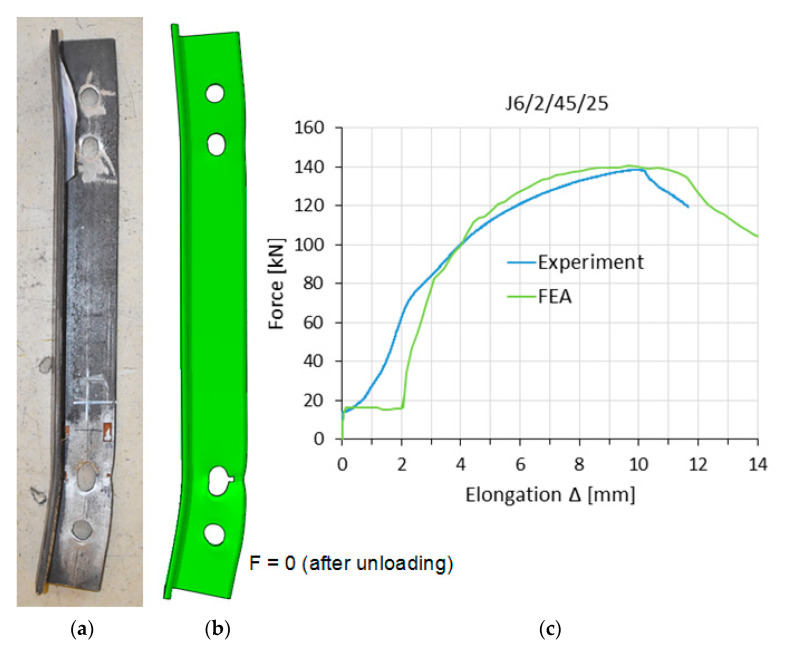
Specimen J6/2/45/25 after fracture: (**a**) experiment, (**b**) FEA model, (**c**) F–Δ curves.

**Figure 9:**
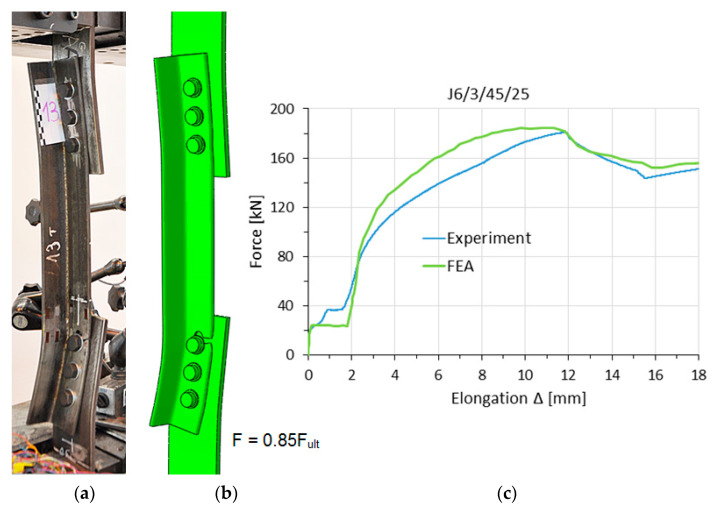
Specimen J6/3/45/25 in the failure phase: (**a**) experiment, (**b**) FEA model, (**c**) F–Δ curves.

Figure 7, Figure 8 and Figure 9 show, for selected elements, a comparison between the numerical models and the experimental tests in the load–elongation measure F–Δ (the displacement difference between reference points). These figures also show obtained global deformation of specimens during experimental and numerical tests at the same load level F.

Table 4 compares the simulation values of the maximum tensile load F_ult,FEM_ and elongation L_ult,FEM_ to the corresponding values from experimental tests (F_ult,Ex_ and L_ult,Ex_). The elongation L_ult,FEM_ (or L_ult,Ex_) refers to the point on the F–Δ curves where the maximum load F_ult,FEM_ (or F_ult,Ex_) was obtained (see Figure 7c). The factors Δ_F_ and Δ_L_ denote the relative difference between the results from simulations and tests, calculated according to Equation (5).

(5) $$\Delta_{F}=\frac{F_{\mathrm{ult},\mathrm{FEA}}-F_{\mathrm{ult},\mathrm{Ex}}}{F_{\mathrm{ult},\mathrm{Ex}}};{\;\Delta }_{L}=\frac{L_{\mathrm{ult},\mathrm{FEA}}-L_{\mathrm{ult},\mathrm{Ex}}}{L_{\mathrm{ult},\mathrm{Ex}}}$$

**Figure 10:**
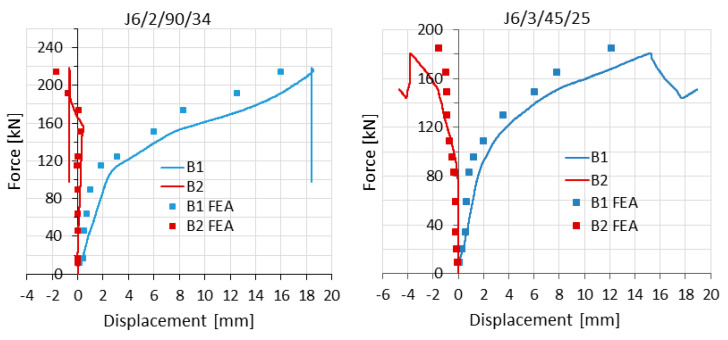
Load–horizontal displacement curves gained from experimental tests and numerical simulations for example elements.

**Figure 11:**
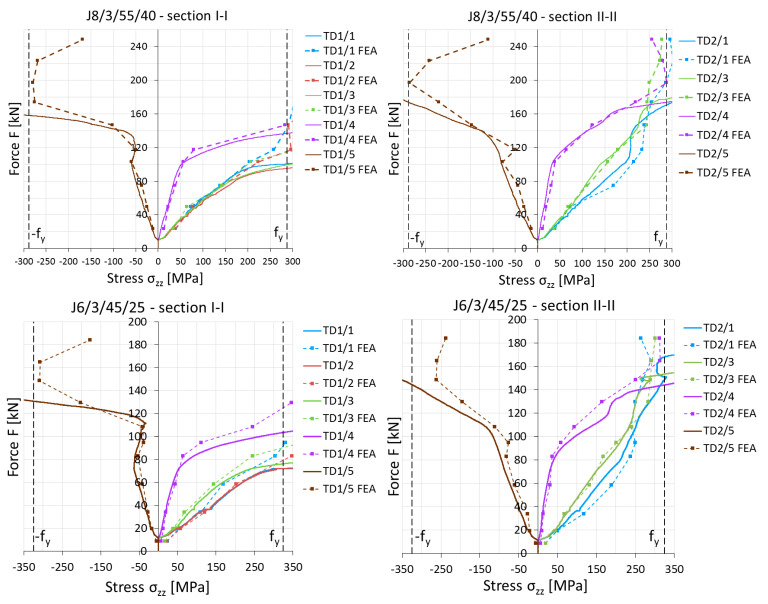
Comparison of longitudinal stress *σ*_zz_ values obtained from experimental tests and numerical simulations for example elements.

For the purpose of validation, measurement results obtained from inductive sensors recording lateral displacements at B1 and B2 (Figure 10) and strain gauges (Figure 11) were also used. For several load levels (from 10% to 100% F_ult,FEM_), appropriate values were obtained from computational models and marked as points on the graphs.

The numerical model predicted the ultimate tensile resistance with high accuracy. The mean value of the relative difference Δ_F_ for the whole group of tested specimens was 0.01 with a standard deviation of 0.03. The predicted global deformation of the elements under load and stress corresponded to the actual connection reactions.

However, the numerical models were characterised by lower elongation compared to the experiments. The mean value of the relative difference Δ_L_ (excluding element J6/2/90/34 for which elongation was measured by machine clamps) was −0.25 with a standard deviation of 0.12. The greater deformation of the test connections compared to those of the FE models can be explained by the influence of the bolt threads (not included in the FE model) and the non-concentric alignment of the bolts and bolt holes in the tested specimens.

## 4. Observed Failure Mechanism

**Figure 12:**
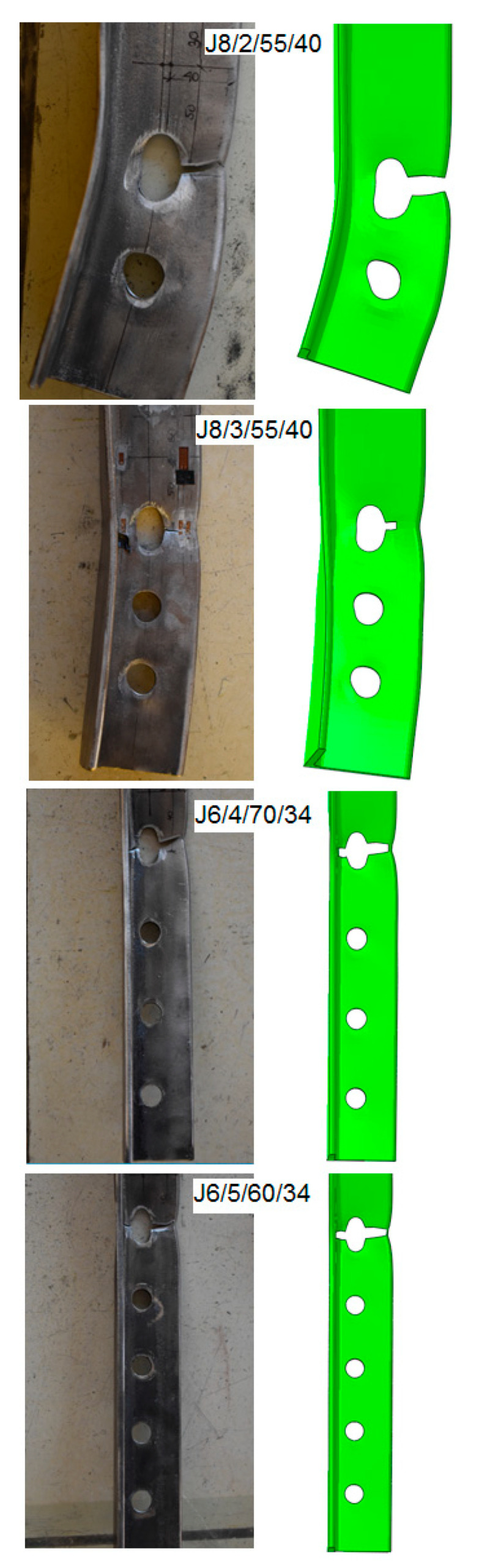
Comparison of failure modes observed in testing and FE modelling.

The final fracture profiles obtained during the FE analyses were very close to those observed during the experiment (Figure 12).

**Figure 13:**
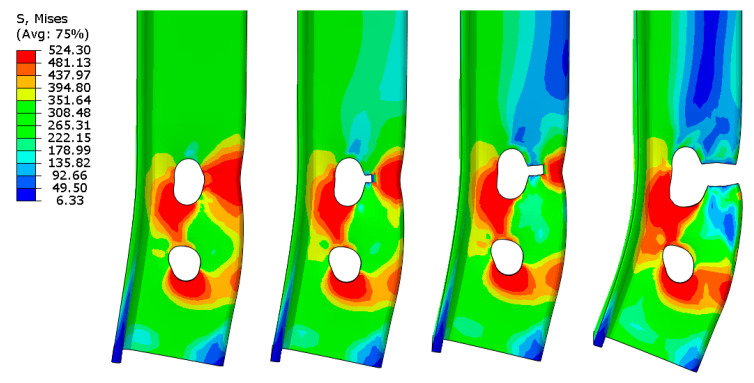
Fracture sequence obtained in FE modelling (specimen J8/2/55/40).

The sequence of crack initiation and propagation obtained in the numerical analyses (shown in Figure 13) was also identical to that observed during the tests.

**Figure 14:**
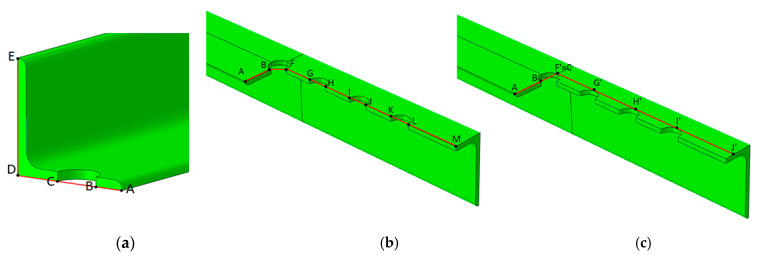
Path definitions: (**a**) in net cross section, (**b**) in block tearing—section along net cross section A_nv_, (**c**) in block tearing—section along gross cross section A_gv_.

However, the ductile fracture propagation and its final position in the angle did not conclusively determine the failure mode. All fractures (in both testing and FE modelling) occurred or started between the bolt hole and the outer edge of the connected leg. This area coincided with the fracture area in block tearing (net area subjected to tension A_nt_) and the net area of the whole angle A_net_, which controls the net cross-section failure. Therefore, to define which failure mode controlled the rupture process, the distribution of effective stresses (σ_eff_, according to the Huber–Mises–Hencky hypothesis) in the net cross-section A_net_ and in the section under block tearing in all specimens was observed. The net cross section was considered along perpendicular lines A–D and D–E across the first internal bolt hole, as shown in Figure 14a. The section subject to block tearing was considered to be line A–B, crossing the net area subject to tension A_nt_, and the line starting from point F and ending in point M, defining the net area subject to shear A_nv_, as shown in Figure 14b. The distribution of effective stress was also considered to be along line A–B, crossing the net area subject to tension A_nt_, and the line between points F’ and J’, defining the gross area subject to shear A_gv_ (Figure 14c).

For all specimens the effective stress σ_eff_ along the A–B section was very similar; the values reached the ultimate strength of the steel. At the level of loading equal to F_ult,FEA_, there was a slight drop in stress on both sides of line A–B after reaching the ultimate strength value. Therefore, only verification of the effective stress distribution along the section subject to shear could distinguish between the failure modes.

**Figure 15:**
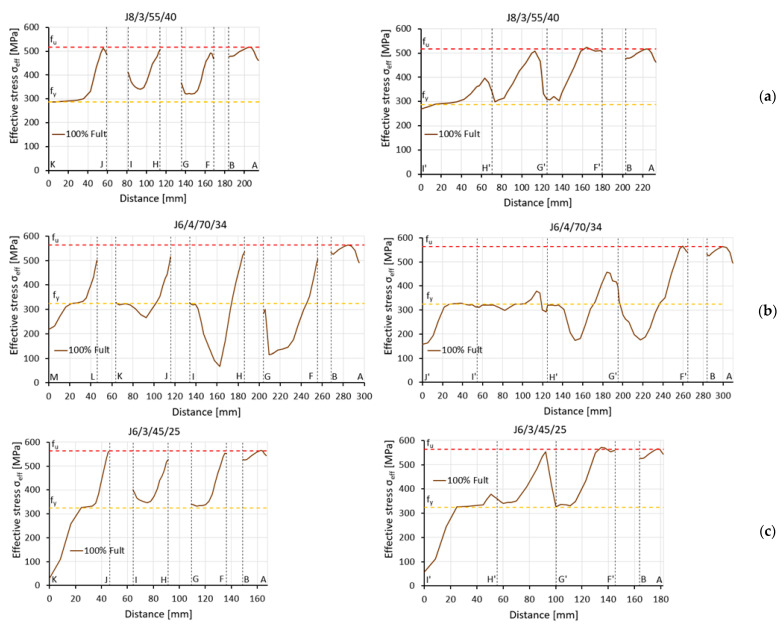
Effective stress distribution in block tearing sections for: (**a**) block tearing, (**b**) net section tearing, (**c**) limited block tearing (left diagrams for net cross section A_nv_, right diagrams for gross cross section A_gv_).

Three different types of effective stress distribution were observed in these areas. In six specimens, effective stresses along the total length of the net cross section F–M or gross cross section F’–J’ achieved or exceeded the yield stress value f_y_, as shown in Figure 15a. This distribution clearly indicated the occurrence of block tearing (BT). For four angles, the effective stresses along F–M or F’–J’ did not fully reach the yield stress f_y_, especially in the area between the bolts, which suggests that net section tearing (NT) was the cause of failure, as shown in Figure 15b. For the other six elements, the effective stress reached the yield stress along the net or gross area subject to shear, but only between the bolts. Along the end distance e_1_ (from the bolt to the adjacent end of the angle, measured in the direction of load transfer), the shear area was not fully yielded. Hence, these elements were classified as subject to a limited block tearing failure mode (BT-L) (Figure 15c).

A comparison between failure modes observed during experimental tests and simulated in analyses (based on the distribution of effective stress along shear area) is shown in Table 5.

**Figure 16:**
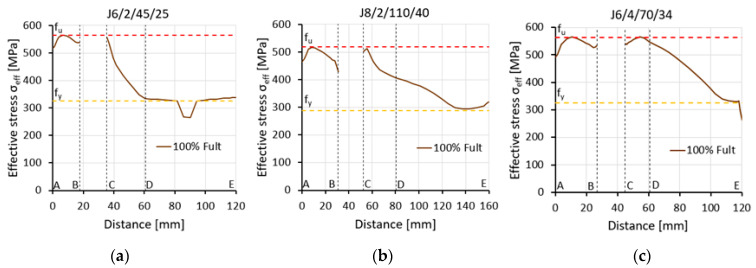
Effective stress distribution in net cross section A_net_ (line A–D) for elements with various failure modes: (**a**) specimen J6/2/45/25 (limited block tearing); (**b**) specimen J8/2/110/40 (block tearing); (**c**) specimen J6/4/70/34 (net section tearing).

There was a very small correlation between the form of fracture observed during the tests and the failure mode determined by the effective stress distribution from the FEA. In addition, effective stress distributions along the net cross-section A_net_ (lines A–E) did not explicitly define the failure mode. However, they were helpful in assessing the conditions in which net section tearing occurred, as shown in Figure 16. The specimens in which net section tearing was predicted by observing the effective stress distribution were characterised by full utilisation of the ultimate resistance of the connected leg and partial utilisation of the ultimate resistance of the outstanding leg (clearly greater than the plastic load capacity). This resulted in high values of the efficiency factor of the net cross section U_eff_ = 0.80–0.91. In the specimens where block tearing or limited block tearing affected the failure mode, full utilisation of the ultimate resistance was reached only in section A–B of the connected leg.

**Figure 17:**
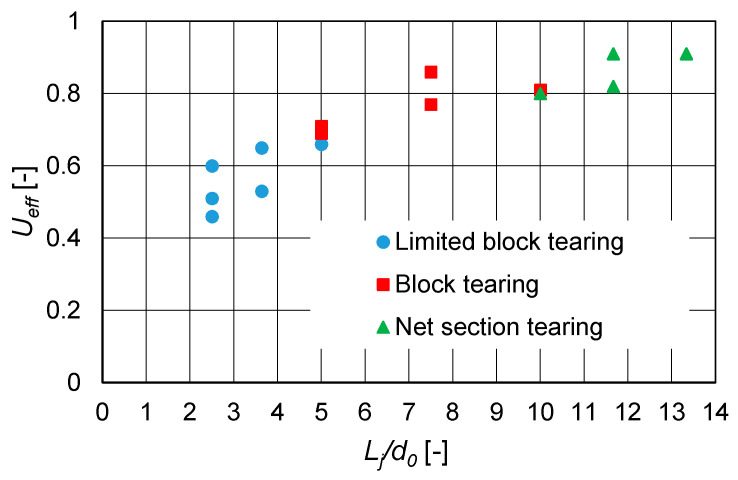
Dependence of efficiency factor U_eff_ and failure mode on relative length of bolted connections L_j_/d_0_.

As shown in Figure 17, it was also observed that the failure mode and efficiency factor of the net cross section U_eff_ were dependent on the relative length of the bolted connections L_j_/d_0_ (where L_j_ is the distance between the centres of the end fasteners in a joint, measured in the direction of force transfer, and d_0_ is the diameter of the bolt hole).

The obtained results suggest that reaching the ultimate resistance of the angle based on net section tearing was possible only in the case of longer joints (L_j_/d_0_ ≥ 10). For shorter connections, the resistance decreased and the angle was subject to block tearing failure. These observations agree with results obtained by other researchers [11,16,17], but the correlation between relative length of bolted connections L_j_/d_0_ and the observed mechanism of block tearing is a new finding. For joints characterised by proportion 5 ≤ L_j_/d_0_ ≤ 10, the shear area could yield along the total length of the net or gross cross section. For very short joints (L_j_/d_0_ ≤ 5), the yielding of the shear area was limited along the end distance e_1_. The obtained ranges of L_j_/d_0_ were relevant to the performed research, but this trend should be further explored in future work.

## 5. Design Considerations

European guidelines for tensioned angles connected by one leg are contained in EN 1993-1-8: Eurocode 3: Design of steel structures—Part 1–8: Design of joints [19]. According to this standard, a single angle in tension connected by a single row of bolts in one leg may be treated as concentrically loaded over an effective net section for which the design ultimate resistance should be determined as follows:

- with one bolt:

(6) $$N_{\mathrm{ult},\mathrm{Rd}}=\frac{2.0\cdot \left(e_{2}-0.5d_{0}\right)\cdot t\cdot f_{u}}{\gamma_{M2}}$$

- with two bolts:

(7) $$N_{\mathrm{ult},\mathrm{Rd}}=\frac{\beta_{2}\cdot A_{\mathrm{net}}\cdot f_{u}}{\gamma_{M2}}$$

- with three or more bolts:

(8) $$N_{\mathrm{ult},\mathrm{Rd}}=\frac{\beta_{3}\cdot A_{\mathrm{net}}\cdot f_{u}}{\gamma_{M2}}$$

where t is the thickness of the connected leg; β_2_ and β_3_ are reduction factors dependent on pitch p_1_; A_net_ is the net area of the angle, and γ_M2_ is a partial factor equal to 1.25.

Eurocode EN 1993-1-8 [19] also requires checking block tearing in lap bolted connections. It distinguishes two cases: for a symmetric bolt group subjected to concentric loading, the use of Equation (9), and for a bolt group subjected to eccentric loading, Equation (10):

(9) $$V_{\mathrm{eff},1,\mathrm{Rd}}=\frac{A_{\mathrm{nt}}\cdot f_{u}}{\gamma_{M2}}+\frac{A_{\mathrm{nv}}\cdot f_{y}}{\sqrt{3}{\;\gamma }_{M0}}$$

(10) $$V_{\mathrm{eff},2,\mathrm{Rd}}=0.5\cdot \frac{A_{\mathrm{nt}}\cdot f_{u}}{\gamma_{M2}}+\frac{A_{\mathrm{nv}}\cdot f_{y}}{\sqrt{3}{\;\gamma }_{M0}}$$

where A_nt_ is the net area subjected to tension, A_nv_ is the net area subjected to shear, and γ_M0_ is a partial factor equal to 1.0. For an angle connected by one leg with a single row of bolts, the stress on the tension area is uniform, and thus Equation (9) is recommended.

Final works on the new version of Eurocode prEN 1993-1-8: 2021 [36] are currently in progress. The proposals for changes include angles connected to one leg. In the new version, the method of calculating the tensile resistance for connections with one bolt does not change; however, for a larger number of fasteners, the tensile resistance should be determined from Equation (11) as the lower value of two: the ultimate tensile resistance of the net cross section and the block tearing resistance computed according to Equation (12) [16,36]:

(11) $$N_{\mathrm{ult},\mathrm{Rd}}=\min\left\{\frac{0.75\cdot A_{\mathrm{net}}\cdot f_{u}}{\gamma_{M2}};{\;V}_{\mathrm{eff},1,\mathrm{Rd}}\right\}$$

(12) $$V_{\mathrm{eff},1,\mathrm{Rd}}=\left[A_{\mathrm{nt}}\cdot f_{u}+\min\left\{\frac{A_{\mathrm{gv}}\cdot f_{y}}{\sqrt{3}};\frac{A_{\mathrm{nv}}\cdot f_{u}}{\sqrt{3}}\right\}\right]/\gamma_{M2}$$

where A_gv_ is the gross area subjected to shear; the rest of variables as in Equations (9) and (10).

**Figure 18:**
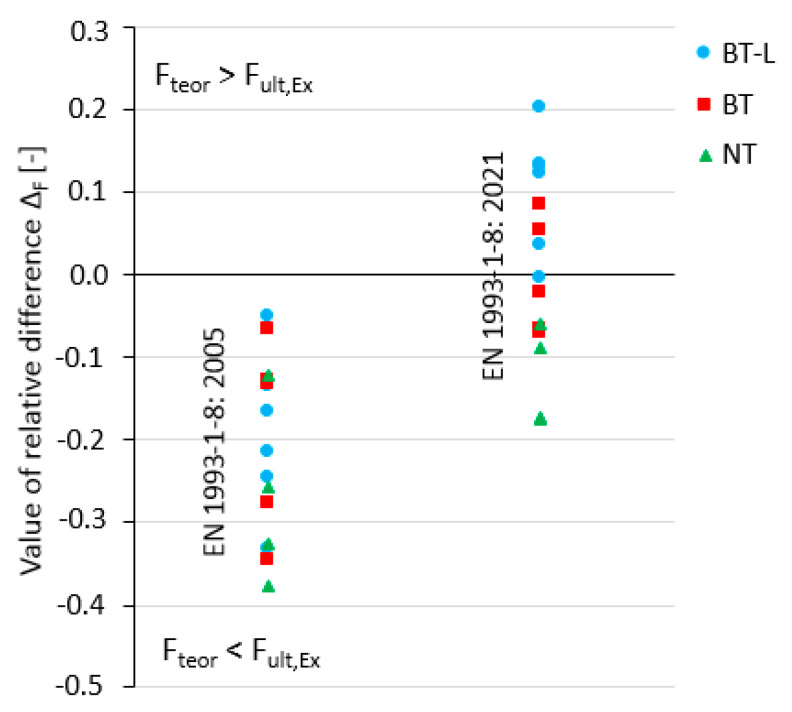
Comparison of relative difference Δ_F_ between maximum tensile load obtained from experimental tests with theoretical resistance calculated according to Eurocode 3 (version from 2005 [19] and new proposal from 2021 [36]).

Figure 18 and Table 6 present a comparison of the maximum tensile load obtained from the experimental test F_ult,Ex_ with the theoretical resistance F_teor_ calculated in accordance with two versions of Eurocode 3 part 1–8: the current version from 2005 [19] and the new draft from 2021 [36]. Using the current Eurocode theoretical resistance, F_teor_ was computed as the minimum value of either the net cross section resistance from Equations (7) or (8), as appropriate according to the number of bolts used, or the block tearing resistance from Equation (9) because of uniform stress distribution along the e_2_ distance. In accordance with the 2021 proposal [36], F_teor_ was computed using Equation (11). All partial factors were assumed to be 1.0. For each test element, except where only bolt failure was observed, values of the relative difference Δ_F_ between F_teor_ and F_ult,Ex_ were calculated (vertical axis in Figure 18), according to Equation (5). The mean values of this parameter Δ_F,m_, as well as its standard deviation Δ_F,s_, minimum Δ_F,min_, and maximum Δ_F,max_ observed values are presented for each edition of the Eurocode in Table 6. If the theoretical resistance *F*_teor_ was limited to Equations (7) or (8) in the case of the current Eurocode [19] or by the first term of Equation (11) in the case of the new Eurocode [36], the letter “N” in Table 6 is next to the Δ_F_ value. If block tearing resistance limited *F*_teor_, there is the indication “V”.

The current version of Eurocode 3 [19] estimates the theoretical resistance on a more conservative level. The mean value of the relative difference Δ_F_ between the theoretical resistance F_teor_ and experimental resistance F_ult,Ex_ was on the level of −0.22. For 13 of the angle elements, the theoretical resistance was determined by net section tearing.

The performance of prEN 1993-1-8 [36] was characterised by a more favourable mean value Δ_F_ parameter equal to 0.0, based on the analyses performed in this study. However, an analysis of Figure 18 shows that, for individual strength functions, compatibility was not so high. For angles where net section tearing occurred, the 2021 draft theoretical resistance calculation underestimated the experimental results. For elements where typical block tearing occurred, the draft theoretical resistance gave very good estimates; the mean value of Δ_F_ was equal to −0.02. However, some angles were destroyed by a failure mode similar to block tearing, but characterised by not fully yielded end distance e_1_ (in this paper classified as subject to limited block tearing failure (BT-L)). In this case, the draft standard theoretical resistance overestimated the experimental results in the range of 0.0–0.20. This failure mode appeared in short connections (L_j_/d_0_ ≤ 5), and the line along which the rupture/yielding occurred was not consistent with typical block tearing, as shown in Figure 13.

## 6. Summary and Conclusions

The failure modes and the tension resistance of angle members connected by one leg with a single row of bolts were investigated experimentally and by means of FEA. Experimental tests indicated a significant influence of the total length of the connection and the edge distance e_2_ on the tensile resistance; however, the obtained results did not allow for the explicit determination of the form of failure (block tearing or net section tearing). Hence, accurate, nonlinear FE models were developed to investigate failure modes. The GTN porous material model was used to analyse the connections through the full scope of the work, from the unloaded state to its rupture.

The main conclusions obtained from the research are as follows:

- The GTN material model prediction showed very high agreement with the results of the experimental tests of the load capacity and global behaviour of the elements.
- The failure modes obtained from FE modelling where GTN material was used agreed to a high degree with the test results regarding to the form of initiation of plastic fracture and its further development.
- Numerical analyses showed three possible failure modes: net-section tearing, typical block tearing and limited block tearing (block tearing with an area not fully sheared).
- This research indicates that, in very short connections (L_j_/d_0_ ≤ 5.0), limited block tearing may determine joint resistance. In this case, full plasticisation does not occur along length e_1_.
- The obtained results of load capacity compared with design procedures given in proposed revisions of Eurocode 3 [36] indicated a satisfactory agreement.
- Nevertheless, analytical models of block tearing did not precisely reflect the actual shear failure path observed in numerical simulations, especially for very short connections where limited block tearing was observed.

Owing to the desire to increase the efficiency of the production of steel structures and to save material, increasingly shorter angle connections are being designed. It seems that limited block tearing (block tearing with an area not fully sheared) is worth further investigation.

## Figures and Tables

**Table 1:** Description of test specimens.

No.	Symbol	Profile	Bolts	p_1_ [mm]	e_1_ [mm]	e_2_ [mm]	Observed Form of Failure	F_ult,Ex_ [kN]	U_eff_ [-]
1.	J8/2/55/40	L80 × 80 × 6	2×M20-10.9	55	70	40	FI	209.8	0.60
2.	J8/2/110/40	L80 × 80 × 6	2×M20-10.9	110	70	40	FP	249.6	0.71
3.	J8/2/55/30	L80 × 80 × 6	2×M20-10.9	55	70	30	FI	163.6	0.46
4.	J8/2/80/40	L80 × 80 × 6	2×M20-10.9	80	70	40	FI	228.5	0.65
5.	J8/2/80/30	L80 × 80 × 6	2×M20-10.9	80	70	30	FI	184.8	0.53
6.	J8/3/55/40	L80 × 80 × 6	3×M20-8.8	55	70	40	FI	242.1	0.69
7.	J8/3/110/40	L80 × 80 × 6	3×M20-8.8	110	70	40	FI	283.3	0.81
8.	J8/4/55/40	L80 × 80 × 6	4×M20-8.8	55	70	40	FP	268.6	0.77
9.	J6/2/45/34	L60 × 60 × 6	2×M16-8.8	45	55	34	BF	179.2	-
10.	J6/2/90/34	L60 × 60 × 6	2×M16-10.9	90	55	34	BF/FI	218.2	0.80
11.	J6/2/45/25	L60 × 60 × 6	2×M16-10.9	45	55	25	FI	138.6	0.51
12.	J6/2/90/25	L60 × 60 × 6	2×M16-8.8	90	55	25	BF	174.0	-
13.	J6/3/45/25	L60 × 60 × 6	3×M16-10.9	45	55	25	FI	180.7	0.66
14.	J6/3/90/25	L60 × 60 × 6	3×M16-10.9	90	55	25	FP	216.7	0.80
15.	J6/4/45/34	L60 × 60 × 6	4×M16-8.8	45	55	34	FP	234.1	0.86
16.	J6/4/70/34	L60 × 60 × 6	4×M16-8.8	70	55	34	FP	245.5	0.91
17.	J6/4/70/25	L60 × 60 × 6	4×M16-8.8	70	55	25	FI	224.1	0.82
18.	J6/5/60/34	L60 × 60 × 6	5×M16-8.8	60	55	34	FP	247.5	0.91

F_ult,Ex_—maximum tensile load registered during single test. BF—bolt failure by shear. FI—tearing from bolt hole to the edge of connected leg. FP—tearing from bolt hole to the edge of connected leg and its propagation towards outstanding leg.

**Table 2:** Material properties.

Element	Yield Strength f_y_ [MPa]		Ultimate Strength f_u_ [MPa]	
	Mean Value	Standard Deviation	Mean Value	Standard Deviation
L80 × 80 × 6	288	3.3	425	4.2
L60 × 60 × 6	325	3.7	470	2.3
Gusset plate	424	5.1	590	4.6

**Table 3:** GTN material parameters introduced to numerical simulations.

*f*_0_	Tvergaard Parameters *q*_i_	*f*_c_	*f*_F_	*f*_N_	*ε*_N_	*s*_N_
0.01	*q*_1_ = 1.5; *q*_2_ = 1.0; *q*_3_ = 2.25	0.06	0.2	0.02	0.3	0.1

**Table 4:** Comparison of tensile resistance and elongation obtained from experimental tests and numerical simulations.

No.	Symbol	F_ult,Ex_ [kN]	F_ult,FEA_ [kN]	Δ_F_ [-]	L_ult,Ex_ [mm]	L_ult,FEA_ [mm]	Δ_L_ [-]
1.	J8/2/55/40	209.8	200.4	−0.04	20.8	14.0	−0.33
2.	J8/2/110/40	249.6	245.9	−0.02	27.3	21.6	−0.21
3.	J8/2/55/30	163.6	158.2	−0.03	15.7	10.4	−0.34
4.	J8/2/80/40	228.5	227.5	−0.004	20.6	18.1	−0.12
5.	J8/2/80/30	184.8	179.6	−0.03	19.8	12.6	−0.37
6.	J8/3/55/40	242.1	248.1	0.02	18.8	15.3	−0.19
7.	J8/3/110/40	283.3	294.4	0.04	21.8	15.3	−0.30
8.	J8/4/55/40	268.6	283.8	0.06	17.7	14.8	−0.16
9.	J6/2/45/34		n.a.	n.a.		n.a.	n.a.
10.	J6/2/90/34	218.2	214.3	−0.02	30.8 *	16.8	n.a.
11.	J6/2/45/25	138.6	140.8	0.02	9.9	9.6	−0.03
12.	J6/2/90/25		n.a.	n.a.		n.a.	n.a.
13.	J6/3/45/25	180.7	184.4	0.02	11.4	10.4	−0.09
14.	J6/3/90/25	216.7	226.7	0.05	16.9	11.5	−0.32
15.	J6/4/45/34	234.1	242.5	0.04	17.0	12.1	−0.29
16.	J6/4/70/34	245.5	255.4	0.04	16.6	11.9	−0.28
17.	J6/4/70/25	224.1	229.1	0.02	15.4	8.7	−0.44
18.	J6/5/60/34	247.5	248.1	0.002	16.4	10.9	−0.34
		Mean value		0.01			−0.25 **
		Standard deviation		0.03			0.12 **

*: displacement measured by machine clamps. **: results excluding J6/2/90/34 element. n.a.: not analysed because of bolt failure.

**Table 5:** Comparison of failure modes obtained from experimental tests and numerical simulations.

No.	Symbol	Failure Mode	
		Test	FE Modelling
1.	J8/2/55/40	FI	BT-L
2.	J8/2/110/40	FP	BT
3.	J8/2/55/30	FI	BT-L
4.	J8/2/80/40	FI	BT-L
5.	J8/2/80/30	FI	BT-L
6.	J8/3/55/40	FI	BT
7.	J8/3/110/40	FI	BT
8.	J8/4/55/40	FP	BT
9.	J6/2/45/34	BF	n.a.
10.	J6/2/90/34	BF/FI	BT
11.	J6/2/45/25	FI	BT-L
12.	J6/2/90/25	BF	n.a.
13.	J6/3/45/25	FI	BT-L
14.	J6/3/90/25	FP	NT
15.	J6/4/45/34	FP	BT
16.	J6/4/70/34	FP	NT
17.	J6/4/70/25	FI	NT
18.	J6/5/60/34	FP	NT

Tests: BF—bolt failure by shear; FI—tearing from bolt hole to the edge of connected leg; FP—tearing from bolt hole to the edge of connected leg and propagation towards outstanding leg. FE modelling: NT—net section tearing; BT—block tearing; BT-L—mixed mode of failure (limited block tearing).

**Table 6:** Values of relative difference Δ_F_ between theoretical resistance F_teor_ and experimental resistance F_ult,Ex_.

No.	Symbol	Failure Mode from FE Model	EN 1993-1-8: 2005		prEN 1993-1-8: 2021	
			Theoretical Resistance Based on:	Δ_F_ [-]	Theoretical Resistance Based on:	Δ_F_ [-]
1.	J8/2/55/40	BT-L	N	−0.33	V	0.0
2.	J8/2/110/40	BT	V	−0.06	N	0.05
3.	J8/2/55/30	BT-L	N	−0.13	V	0.12
4.	J8/2/80/40	BT-L	N	−0.17	V	0.04
5.	J8/2/80/30	BT-L	V	−0.05	V	0.13
6.	J8/3/55/40	BT	N	−0.28	N	0.09
7.	J8/3/110/40	BT	N	−0.13	N	−0.07
8.	J8/4/55/40	BT	N	−0.35	N	−0.02
9.	J6/2/45/34	n.a.	-	-	-	-
10.	J6/2/90/34	BT	N	−0.13	N	−0.07
11.	J6/2/45/25	BT-L	N	−0.21	V	0.20
12.	J6/2/90/25	n.a.	-	-	-	-
13.	J6/3/45/25	BT-L	N	−0.24	N	0.13
14.	J6/3/90/25	NT	N	−0.12	N	−0.06
15.	J6/4/45/34	BT	N	−0.42	N	−0.13
16.	J6/4/70/34	NT	N	−0.32	N	−0.17
17.	J6/4/70/25	NT	N	−0.26	N	−0.09
18.	J6/5/60/34	NT	N	−0.38	N	−0.17
			Δ_F,m_	−0.22		0.00
			Δ_F,s_	0.11		0.12
			Δ_F,min_	−0.42		−0.17
			Δ_F,max_	−0.05		0.20

## Data Availability

Data is contained within the article.
